# *In Utero* Administration of Drugs Targeting Microglia Improves the Neurodevelopmental Outcome Following Cytomegalovirus Infection of the Rat Fetal Brain

**DOI:** 10.3389/fncel.2018.00055

**Published:** 2018-03-06

**Authors:** Robin Cloarec, Sylvian Bauer, Natacha Teissier, Fabienne Schaller, Hervé Luche, Sandra Courtens, Manal Salmi, Vanessa Pauly, Emilie Bois, Emilie Pallesi-Pocachard, Emmanuelle Buhler, François J. Michel, Pierre Gressens, Marie Malissen, Thomas Stamminger, Daniel N. Streblow, Nadine Bruneau, Pierre Szepetowski

**Affiliations:** ^1^INMED, French National Institute of Health and Medical Research INSERM U1249, Aix-Marseille University, Marseille, France; ^2^Neurochlore, Marseille, France; ^3^French National Institute of Health and Medical Research INSERM U1141, Paris Diderot University, Sorbonne Paris Cité, Paris, France; ^4^PremUP, Paris, France; ^5^PPGI Platform, INMED, Marseille, France; ^6^Centre National de la Recherche Scientifique CNRS UMS3367, CIPHE (Centre D'Immunophénomique), French National Institute of Health and Medical Research INSERM US012, PHENOMIN, Aix-Marseille University, Marseille, France; ^7^Laboratoire de Santé Publique EA 3279, Faculté de Médecine Centre d'Evaluation de la Pharmacodépendance-Addictovigilance de Marseille (PACA-Corse) Associé, Aix-Marseille University, Marseille, France; ^8^PBMC platform, INMED, Marseille, France; ^9^InMAGIC platform, INMED, Marseille, France; ^10^Institute for Clinical and Molecular Virology, University of Erlangen-Nuremberg, Erlangen, Germany; ^11^Vaccine and Gene Therapy Institute, Oregon Health and Science University, Portland, OR, United States

**Keywords:** cytomegalovirus, microglia, rat model, fetal brain, neurological outcome

## Abstract

Congenital cytomegalovirus (CMV) infections represent one leading cause of neurodevelopmental disorders. Recently, we reported on a rat model of CMV infection of the developing brain *in utero*, characterized by early and prominent infection and alteration of microglia—the brain-resident mononuclear phagocytes. Besides their canonical function against pathogens, microglia are also pivotal to brain development. Here we show that CMV infection of the rat fetal brain recapitulated key postnatal phenotypes of human congenital CMV including increased mortality, sensorimotor impairment reminiscent of cerebral palsy, hearing defects, and epileptic seizures. The possible influence of early microglia alteration on those phenotypes was then questioned by pharmacological targeting of microglia during pregnancy. One single administration of clodronate liposomes in the embryonic brains at the time of CMV injection to deplete microglia, and maternal feeding with doxycyxline throughout pregnancy to modify microglia in the litters' brains, were both associated with dramatic improvements of survival, body weight gain, sensorimotor development and with decreased risk of epileptic seizures. Improvement of microglia activation status did not persist postnatally after doxycycline discontinuation; also, active brain infection remained unchanged by doxycycline. Altogether our data indicate that early microglia alteration, rather than brain CMV load *per se*, is instrumental in influencing survival and the neurological outcomes of CMV-infected rats, and suggest that microglia might participate in the neurological outcome of congenital CMV in humans. Furthermore this study represents a first proof-of-principle for the design of microglia-targeted preventive strategies in the context of congenital CMV infection of the brain.

## Introduction

Perinatal and congenital infections cause morbidity and mortality throughout the world. Some pathogens are of considerable public health impact, such as *Toxoplasma gondii*, rubella, human immunodeficiency virus, Zika virus, and human cytomegalovirus (CMV). CMVs belong to the *Herpesviridae* family. In humans, congenital CMV infection can cause severe neurological diseases and defects (Adler and Nigro, 2013). These include microcephaly, polymicrogyria, hearing loss, cerebral palsy, epileptic seizures and intellectual disability, as well as the as-yet elusive influence on the emergence of schizophrenia, autism or epilepsy. Despite the incidence and the medical and socioeconomical burden of congenital CMV, which represents about 1% of all live births, the pathophysiological mechanisms underlying the emergence of neurodevelopmental disorders remain elusive (Cheeran et al., 2009). In the absence of effective preventive or curative therapies, understanding the pathogenesis is mandatory before strategies for early interventions can be designed and tested. The pathophysiology of congenital CMV disease is inherently complicated and involves different stages, from maternal CMV primary infection or reactivation and the associated maternal immune responses, to infection and dissemination within the developing brain—not to mention the crossing of the placental and blood-brain barriers.

Insights into the early events following CMV infection of the developing brain are particularly needed. CMVs are generally species-specific; thus, the development of relevant animal models has been, and will continue to be, critical to our understanding of the mechanisms involved in CMV congenital brain disease (Britt et al., 2013; Cekinovic et al., 2014). Whereas multiple routes (intracranial, intraperitoneal or intraplacental) and developmental timepoints (antenatal or neonatal) of CMV inoculation were used, and despite the lack of materno-fetal transmission of CMV infection in rodents, convergent insights into the alteration of innate and adaptive immune responses have emerged from such models (Kosmac et al., 2013; Sakao-Suzuki et al., 2014; Bradford et al., 2015; Slavuljica et al., 2015; Cloarec et al., 2016; Seleme et al., 2017). The production of cytokines by glial cells, the recruitment of peripheral immune cells, and the altered status of microglia, are all likely to influence neuropathogenesis. Microglia are targeted by CMV during human congenital disease (Teissier et al., 2014) and in murine models of intraplacental or neonatal infections (Kosugi et al., 2002; Sakao-Suzuki et al., 2014). Recently, we reported on a rat model of CMV infection of the developing brain displaying prominent infection of brain myelomonocytic cells and early alteration of microglia (Cloarec et al., 2016). Microglial cells originate from erythromyeloid progenitors located in the yolk sac during embryogenesis (Ginhoux et al., 2010) and represent the resident mononuclear phagocytes of the brain (Ginhoux et al., 2013; Ginhoux and Jung, 2014). These cells play crucial roles not only in immune defense, maintenance of the neural environment, injury, and repair, but also in neurogenesis, synaptogenesis, synaptic pruning, connectivity, and modulation of synaptic and neuronal activity (Frost and Schafer, 2016). Importantly, early microglial responses might well combat against CMV infection; but these responses might likely have detrimental effects by interacting with important neurodevelopmental processes.

To which extent and to which direction—favorable or detrimental—early microglia alteration would influence the emergence and severity of neurodevelopmental phenotypes in the developing brain *in utero* in the context of CMV infection represent an important pathophysiological question. Herein, we have tested whether early pharmacological targeting of microglia during pregnancy impacts postnatal neurological manifestations in our previously reported rat model of CMV infection of the embryonic brain (Cloarec et al., 2016) and have identified a critical role for microglia.

## Materials and methods

### Experimental design

In this study, we explored whether neuroimmune events associated with brain CMV infection *in utero* could be involved in the emergence of postnatal neurological consequences. We first explored whether infected rats would display phenotypes related to the human pathology. We then tested two independent methods in order to target microglia: (1) doxycycline treatment, which is known to attenuate microglia activation in the developing brain (Cunningham et al., 2013) and (2) liposomes containing clodronate to deplete microglia by uptake and release into the cytosol of a non-hydrolysable ATP analog leading to cell death. Finally, we determined whether animals would still display postnatal neurological consequences following each treatment.

### Ethical statement

Animal experimentations were performed in accordance with the French legislation and in compliance with the European Communities Council Directives (2010/63/UE). Depending on the age of the animals, euthanasia were performed after anesthesia with 4% isoflurane with overdose of pentobarbital (120 mg/kg) or with decapitation. This study was approved under the French department of agriculture and the local veterinary authorities by the Animal Experimentation Ethics Committee (*Comité d'Ethique en Expérimentation Animale)* n°14 under licenses n°01010.02 and n°2016100715494790.

### CMV infection and pharmacological treatments

Wistar rats (Janvier Labs, France) were raised and mated at INMED Post Genomic Platform (PPGI) animal facility. Rat CMV recombinant Maastricht strain (RCMV-Δ145-147-gfp) with a green fluorescent protein (GFP) expression cassette, and its production, purification and titration, were reported previously (Baca Jones et al., 2009). *In utero* intracerebroventricular (icv) injections were performed at embryonic day 15 (E15) as previously described (Salmi et al., 2013; Cloarec et al., 2016) in embryos from timed pregnant rats that were anaesthetized with ketamine (100 mg/kg)/xylazine (10 mg/kg). Microglia were depleted *in vivo* with clodronate liposomes icv (0.5 μL/injection, Encapsula Nanosciences) co-injected with 1.75 10^3^ pfu of rat CMV; alternatively, phosphate-buffered saline (PBS)-containing liposomes (0.5 μL/injection) were co-injected as a control (untreated condition). Microglia status was modified in the embryos *in vivo* with doxycycline *per os* given to the mother throughout pregnancy (200 mg/kg in food pellet chow, Safe).

### Immunohistochemistry experiments

Immunohistochemistry experiments on coronal brain sections (50–100 μm, vibratome, Microm; 14 μm, cryostat, Leica) were carried out as described previously (Cloarec et al., 2016) with the following primary (anti-Iba1: 1/500, Wako; anti-Cd68, Ed1 clone: 1/200, Millipore) and secondary (Alexa Fluor 568 or 647-conjugated goat anti-rabbit or anti-mouse IgGs; Life Technologies) antibodies. Hoescht 33258 (1:2000, Sigma) was used for nuclei staining.

For tissue clearing experiments (see next subsection), whole infected brains were first immunostained as follows. Tissue samples were dehydrated in methanol/1X PBS series: 20, 40, 60, 80, 100 × 2 for 1h each at room temperature (RT) and then incubated overnight at RT on a platform shaker in a solution of PBSG-T [PBS 1X containing 0.2% gelatin (Sigma-Aldrich), 0.5% Triton X-100 (Sigma-Aldrich) and 0.02% Sodium-Azide (Sigma-Aldrich)]. Next, samples were transferred to PBSG-T containing anti-GFP antibodies (AVES, 1:2,000) and placed at 37°C, with rotation at 100 rpm, for 10 days. This was followed by six washes of 1 h in PBSG-T at RT. Samples were then incubated in secondary antibodies (Donkey anti-chicken Alexa-Fluor 647, Jackson ImmunoResearch, 1:500) diluted in PBSG-T for 2 days at 37°C. After six washes of 1 h in PBSG-T at RT, samples were stored at 4°C in PBS until clearing.

### Tissue clearing

Tissue clearing was performed according to previously reported 3DISCO and iDISCO+ clearing procedures (Erturk et al., 2012; Belle et al., 2014, 2017; Renier et al., 2014) with slight modifications. Briefly, all incubation steps were performed at RT in a fume hood using a 15 ml centrifuge tube (TPP, Dutscher). Samples were first dehydrated in a graded series (20, 40, 60, 80, and 100%) of methanol (Sigma-Aldrich) for 1 h. This was followed by a delipidation step of 20 min in dichloromethane (DCM; Sigma-Aldrich). Samples were transferred to 100% DCM and finally cleared overnight at RT in dibenzylether (DBE; Sigma-Aldrich).

### Quantitative reverse transcription polymerase chain reaction (qRT-PCR)

Total RNA samples were extracted from whole CMV-infected brains at P1 using TRIZOL reagent (Life Technology). cDNA was synthesized from 1 μg of total RNA using Quantitect Reverse Transcription Kit according to manufacturer protocol (Qiagen). RT-PCRs were carried out using SYBR-Green chemistry (Roche Diagnostics) and Roche amplification technology (Light Cycler 480). PCR primers were designed for GFP transcripts (forward: 5′-gggcacaagctggagtaca; reverse: 5′-cttgatgccgttcttctgc) and for control gene *Rpl19* (ribosomal protein L19) (Cloarec et al., 2016). Primer pairs were optimized to ensure specific amplification of the PCR product and the absence of any primer dimer. Quantitative PCR standard curves were set up for all analyses.

### Microscopy, cell counting, and image analyses

Images of brain sections were acquired with a Stereo Microscope Olympus SZX16 equipped with digital camera DP73, or a Zeiss Axio Imager Z2 microscope with structured illumination (ApoTome) equipped with Zeiss AxioCam MRm camera and processed using Axiovision software, or with a confocal laser scanning microscope Leica TCS SP5X equipped with a white light laser, a 405 nm diode for ultra-violet excitation, and 2 HyD detectors. For cell counting analyses on immunostained brain sections, at least three adjacent brain sections were analyzed throughout the entire z-dimension for each sample using confocal microscopy, according to previously reported procedures (Cloarec et al., 2016) and as further detailed in Supplementary Materials. A phagocytic activation index (PAI) was defined as the ratio of Iba1^+^ Ed1^+^ cells to the total number of Iba1^+^ cells. ImageJ software was used to quantify fluorescence areas on coronal brain sections selected according to their coordinates, as indicated in the rat brain atlas (Khazipov et al., 2015), excluding the meninges. Percentage of fluorescence area of a given brain section was obtained by normalizing to the total area of this brain section.

Whole-brain imaging after tissue clearing was performed using ImspectorPro software (LaVision BioTec) with a binocular stereomicroscope (MXV10, Olympus) equipped with a 2X objective (MVPLAPO, Olympus) used at magnifications 0.8X. A laser (NKT Photonics SuperK extrem) with a 640/30 nm emision filtre and two cylindrical lenses was used to generate a light sheet. Samples were placed in an imaging chamber made of 100% quartz (LaVision BioTec) filled with DBE and illuminated from the side by the laser. Images were acquired with an Andor NEO sCMOS camera (2,560 × 2,160 pixel size, LaVision BioTec). The Z-step size between each image was fixed at 3 μm. 3D (three-dimensional)-images, quantifications and movies were generated using Imaris x64 software (version 8.4.1, Bitplane). Stack images were first converted to Imaris file (.ims) using ImarisFileConverter. 3D reconstruction of the sample was performed using “volume rendering.” Brain segmentation was performed using “surface” tool by creating a mask around each volume to remove meninges and peripheral tissue containing antibody aggregates, allowing GFP intensity quantification only in brain parenchyma. GFP mean intensity obtained was then normalized to the total volume of segmented brain. 3D pictures and movies were generated using the “snapshot” and “animation” tools.

### Flow cytometry

Leukocytes from brains obtained from anesthetized P1 or P7 pups were isolated as previously described (Cloarec et al., 2016). Approximately, 1–3 × 10^6^ leukocytes were incubated with Zombie NIR Fixable Viability kit (1:200; Biolegend) for 20 min at RT. Fc receptors were blocked using mouse anti-rat CD32 antibody (FcγII receptor, clone D34-485) for 10 min. at 4°C to reduce nonspecific binding. Blocked cell samples were stained with antibodies against combinations of cell surface markers as previously described (Cloarec et al., 2016). An average of 1.3 × 10^5^ living singlet cells were analyzed per brain equivalent on a BD LSRFortessa cell cytometer and raw data were analyzed using FACSDiva V8.0 software (BD Biosciences).

### Phenotyping

Acquisition of classical developmental righting and cliff aversion reflexes was monitored daily between postnatal day 1 (P1) and P20 as further detailed in Supplementary Materials. The presence of hindlimb paralysis was determined visually in animals that had a postural misplacement and immobility of their hindlimbs. Generalized tonic-clonic epileptic seizures (GTCS) were detected visually, usually after animal handling, especially during cage changing and behavioral testing. They consisted in a classical behavioral sequence including (i) movement arrest and loss of postural equilibrium (ii) hypertonic posture of the trunk, limbs and tail, symmetrically, and (iii) repeated, large clonic movements of all limbs, often with respiratory arrest, incontinence, motor automatisms such as chewing and grooming, terminated by a catatonic phase. Auditory experiments were performed and monitored as detailed in Supplementary Figure 1.

### Data analysis and statistics

Data were expressed as means ± s.e.m. unless otherwise stated. Non-parametric Mann Whitney test (two-tailed) followed by Bonferroni correction, if needed, and non-parametric Kruskall-Wallis test were used to detect heterogeneous distribution between groups followed by Dunn's post-hoc test for multiple comparisons. Univariate Cox analysis and Fisher's exact test (two-tailed) were used to compare between survival distributions and rates. Parametric Student's *t*-test was used to compare between body weight gains, whereas Chi-square test with Bonferroni correction and mixed model for repeated data were used for all other phenotypic comparisons between groups of animals. Generalized mixed models allow incorporating correlations while observations are collected over time. The group of animal was entered as a covariate and the time point (time when measures were repeated) was introduced as a random effect with autogressive covariance structure to account for the within-subjects correlations, assuming that two measurements timely close to each other are closely correlated, and less correlated when they get farther apart. As we are modeling binary data, we specified the binomial distribution and the logit function as the link one using the PROC GLIMMIX with sas 9.4. Significance threshold was set at 0.05 unless otherwise stated in the figure legends.

## Results

### CMV infection of the rat fetal brain leads to postnatal mortality and to neurological manifestations

To determine whether CMV infection and the accompanying immune responses in the developing rat brain could be associated with the emergence of postnatal consequences, recalling those seen in the corresponding human congenital disorder, recombinant rat CMV expressing GFP was injected icv in embryos from timed-pregnant rats at E15 as previously described (Cloarec et al., 2016).

**Figure 1:**
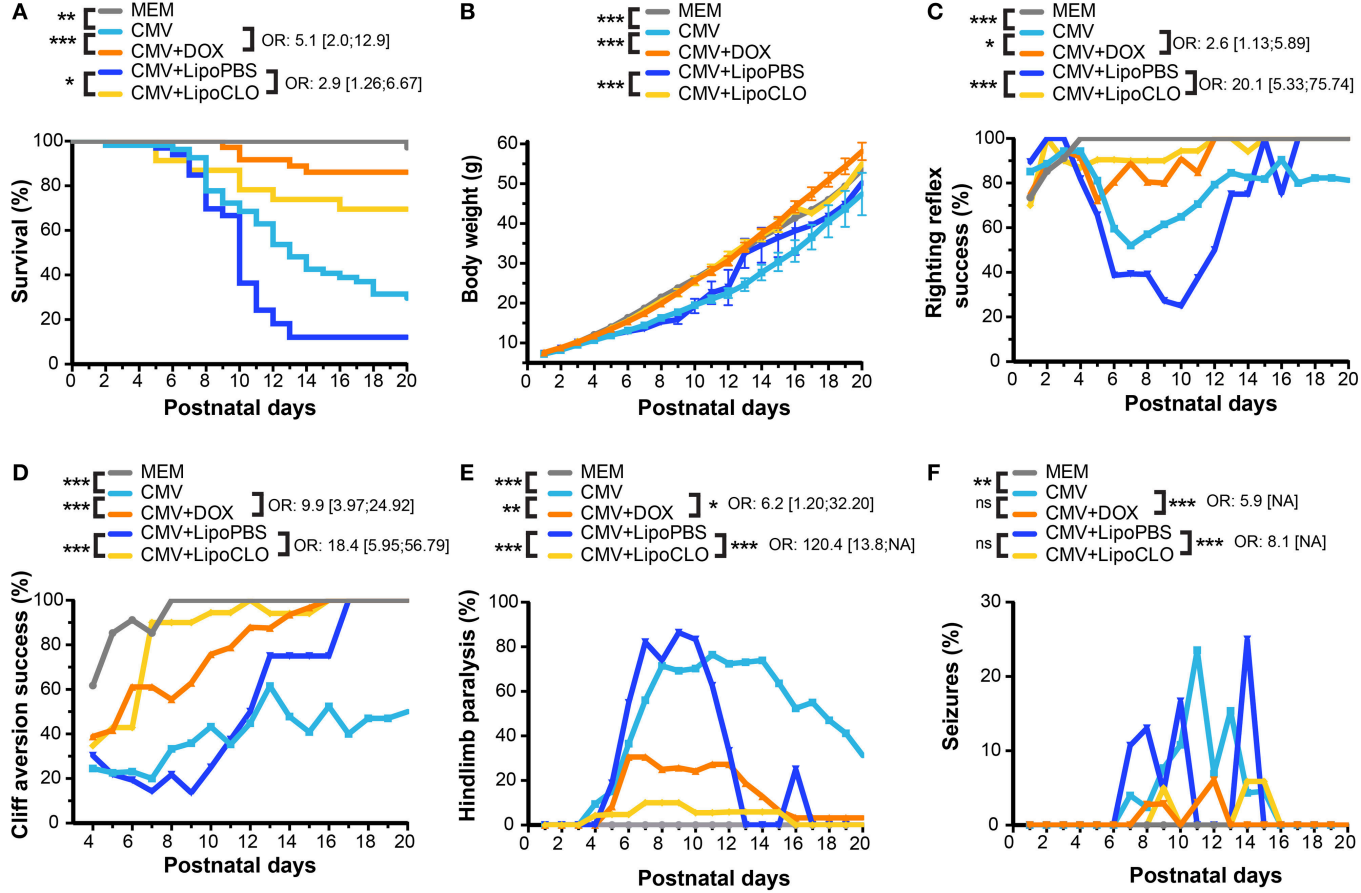
CMV infection of the embryonic rat brain causes decreased survival and neurodevelopmental defects that can be prevented *in utero* by microglia-targeted drug-based strategies. Phenotypic investigations (see Figure 2) in the three first postnatal weeks in pups previously subjected to the following procedures during pregnancy: vehicle intraventricularly (icv) (MEM; *n* = 34, four litters); CMV icv, pregnant rat fed with doxycycline (DOX) during pregnancy (CMV+DOX; *n* = 36, four litters); CMV icv, untreated pregnant rat (CMV; *n* = 54, six litters); CMV with clodronate liposomes icv (CMV+LipoCLO; *n* = 23, two litters); CMV with PBS liposomes icv (CMV+LipoPBS; *n* = 33, four litters). Sex ratio did not differ between the five groups at birth (*p* = 0.15, Chi-square test). **(A)** Kaplan-Meier survival curves indicated significant decreased survival in the CMV vs. MEM group, and significant rescues in the CMV+DOX and CMV+LipoCLO groups vs. their untreated counterparts (CMV and CMV+LipoPBS, respectively). **(B)** Cumulative body weight decreased significantly in the CMV vs. MEM group. Significant improvements were seen in the CMV+DOX and CMV+LipoCLO groups vs. their untreated counterparts (CMV and CMV+LipoPBS, respectively). **(C,D)** The proportion of animals succeeding to the righting reflex **(C)** or the cliff aversion reflex **(D)** sensorimotor tests decreased significantly in the CMV vs. MEM group, and significantly improved in the CMV+DOX vs. CMV group, and in the CMV+LipoCLO vs. CMV+LipoPBS group. **(E,F)** Hindlimb paralysis **(E)** and generalized tonic-clonic epileptic seizures (GTCS) **(F)** occurred significantly more frequently in the CMV vs. MEM group. This improved significantly in the CMV+DOX vs. untreated CMV group and in the CMV+LipoCLO vs. untreated CMV+LipoPBS group. Note that because the most severely affected pups were at higher risk to death, a misleading effect of apparent improvement with time could be perceived from curve shapes even for untreated conditions. Left sides of the cohorts legend: univariate Cox analysis **(A)**, mixed model for repeated data **(B–D)**, or Chi2 test with Bonferroni correction **(E,F)**. Right sides of the cohorts legend: odds ratio (OR) ± confidence intervals correspond to the risks in the untreated vs. their corresponding treated groups: postnatal mortality **(A)**; failure at test **(C,D)**; hindlimb paralysis **(E)**; GTCS **(F)**. ***p < 0.001; **p < 0.01; *p < 0.05; ns, not significant, for all figure panels, except **(E,F)** where ***p < 0.0002; **p < 0.002. NA, not available.

**Figure 2:**
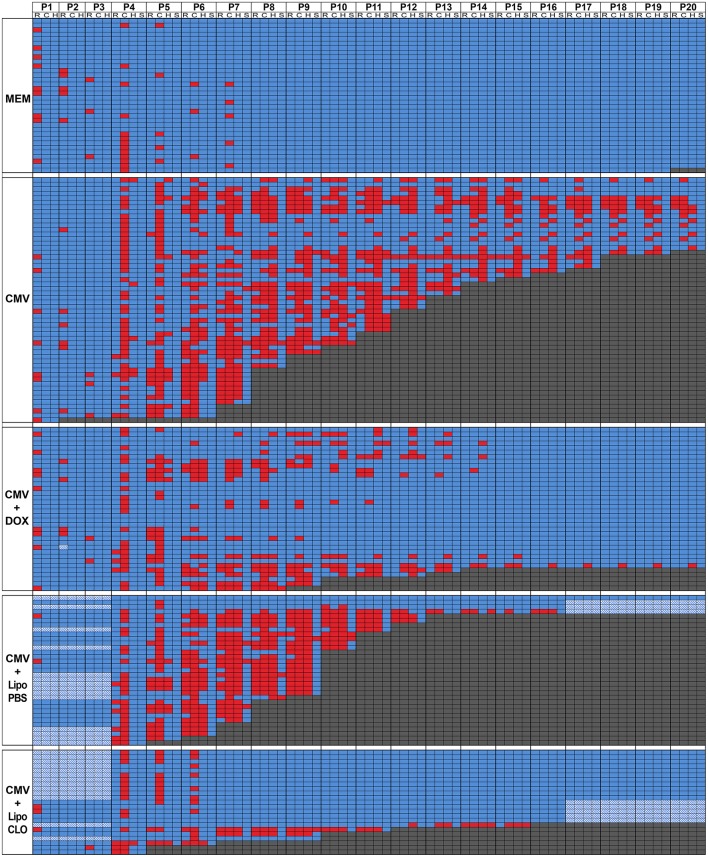
Overview of day-per-day phenotype assessment in the first three postnatal weeks. Color-encoded overview of postnatal test evaluations and observations performed as from postnatal day P1 (see also Figure 1; Supplementary Table 1). Five experimental cohorts of rats were analyzed postnatally: i/control embryos were injected intraventricularly (icv) with vehicle at E15 (MEM; *n* = 34 from four litters); ii/embryos were injected icv with rat CMV at E15 and were born from dams fed with control chow all over pregnancy (CMV; *n* = 54 from six litters); iii/embryos were injected icv with rat CMV at E15 and were born from dams fed with doxycycline-supplemented chow all over pregnancy (CMV+DOX; *n* = 36 from four litters); iv/embryos were injected icv with rat CMV and with control (PBS) liposomes at E15 (CMV+LipoPBS; *n* = 33 from 4 litters); v/embryos were injected icv with rat CMV and with clodronate liposomes at E15 (CMV+LipoCLO; *n* = 23 from 2 litters). The table shows color-encoded results for each individual pup evaluated on a daily basis (P1 to P20 columns) for the phenotypic parameters as detailed below. Performances at the righting (R sub-column) and cliff aversion (C sub-column) reflexes were measured from P1 or from P4, respectively; performances to right (R) or to turn away from the cliff (C) were color-encoded following a binary rule (blue: success; red: failure). For righting reflex evaluation, rat pups were placed in a supine position, and the time required to flip to the prone position was measured. For the cliff aversion reflex, animals were placed with their forepaws overhanging the edge of a board; the time required to turn >90° away from the edge was recorded. For both tests, a maximum observation time of 30 s. was used. Each pup was also monitored daily for the appearance of hindlimb paralysis (H sub-column) and of generalized tonic-clonic seizures (GTCS) (S sub-column); the data were color-encoded following a binary rule where blue reflected the absence of either event, whereas red indicated the occurrence of paralysis or seizure. When pups died, this was also annotated on the table and color-encoded in dark gray. Hatched cells: data unavailable.

During the period of evaluation, i.e., until P20, CMV infection significantly decreased postnatal survival as compared with control animals, which were injected icv with the vehicle (MEM) (*p* = 0.0017) (Figures 1A, 2; Supplementary Table 1). Indeed, 70.4% (38 out of 54 pups) of CMV-infected newborns died in the first three postnatal weeks, compared to 2.9% (one out of 34) controls (*p* < 10^−4^, Chi2 test). In contrast, antenatal mortality was not affected as no significant difference was observed in the ratio of live animals at birth (72.8%, *n* = 103) as compared to controls (68.5%, *n* = 54) (Fisher's exact test, two-tailed). Body weight was similar at P1 between CMV-infected (7.20 g ± 0.10) and control (7.40 g ± 0.11) newborns (Student's *t*-test), but CMV infection *in utero* significantly impacted the postnatal evolution of body weight gain (*p* < 10^−4^) (Figure 1B; Supplementary Table 1).

Congenital CMV infection is the leading cause of non-hereditary congenital sensorineural hearing loss (Smith et al., 2005). In order to assess whether CMV infection *in utero* causes hearing loss in rats, auditory tests were performed at P40. Hearing thresholds were significantly higher for clicks (*p* = 0.0146) and for 24 kHz bursts (*p* = 0.0017) in the CMV infected group compared to the control group (Supplementary Figure 1; Supplementary Table 1). As hearing loss caused by congenital CMV infection may be progressive in children, we aimed at evaluating hearing thresholds in CMV-infected animals at later ages. However, a significant deterioration of hearing thresholds was also detected in control (MEM-injected) rats between P40 and P60 (data not shown); this restrained us from performing such a longitudinal analysis in CMV-infected rats as the deterioration of hearing thresholds seen in control rats would likely preclude reliable interpretation of the overall data.

Human congenital CMV infection is also well known to be a contributing cause of cerebral palsy, a group of disorders involving variable degree of sensorimotor disabilities (Colver et al., 2014). Sensorimotor development was evaluated by daily monitoring pups between P1 and P20 for the acquisition of the classic righting and cliff aversion reflexes (Rousset et al., 2013). The righting reflex consisted in assessing the ability of rodent pups to coordinate the necessary movement to roll over from their backs onto their paws. A significant proportion of CMV-infected pups showed inability to right, as compared with MEM-injected pups, which all performed the test successfully (*p* < 10^−4^; Figures 1C, 2; Supplementary Figure 2A; Supplementary Table 1). In the cliff aversion test, pups were placed with their forepaws overhanging the edge of a board and the time required to turn away from the edge was recorded. Control MEM-injected rats showed a clear performance improvement from P4 as all succeeded the test after P8 (Figures 1D, 2; Supplementary Table 1). In contrast, a significant proportion of CMV-infected pups was unable to complete the cliff aversion test all along the first three postnatal weeks (*p* < 10^−4^; Figure 1D, 2; Supplementary Figure 2B; Supplementary Table 1). Hindlimb paralysis was detected in the first three postnatal weeks in 83.3% of CMV-infected pups, likely preventing animals from turning away from the cliff. Hindlimb paralysis was not seen in any non-infected pup (*p* < 10^−4^; Figures 1E, 2; Supplementary Table 1).

Patients with human congenital CMV are also at high risk of postnatal epileptic seizures (Suzuki et al., 2008). Consistently, 24.1% of CMV-infected rat pups exhibited GTCS at least once in the first three postnatal weeks whereas none of MEM-injected pups ever showed any GTCS (*p* = 0.002; Figure 1F, 2; Supplementary Table 1). Interestingly, a clear relationship between epileptic seizures and death was observed wherein 85% of seizing rats deceased, as compared to 37% of non-seizing rats (*p* < 10^−4^, Chi2 test). Similarly, 72% of rats with hindlimb paralysis deceased during the period of evaluation, as compared to 13% of non-paralyzed rats (*p* < 10^−4^).

### Acute icv injection of clodronate liposomes *in utero* depletes microglia and improves the postnatal outcome

The consequences of rat CMV infection as described above, recapitulated several cardinal clinical features of the human pathology. The early and prominent infection and alteration of microglia upon CMV infection of the developing rat brain *in utero* (Cloarec et al., 2016) suggested that microglia might contribute to the pathophysiology of congenital CMV. In order to evaluate the possible impact of microglia on the emergence of the neurological and other developmental defects, experiments were designed to target microglia with drugs during pregnancy.

**Figure 3:**
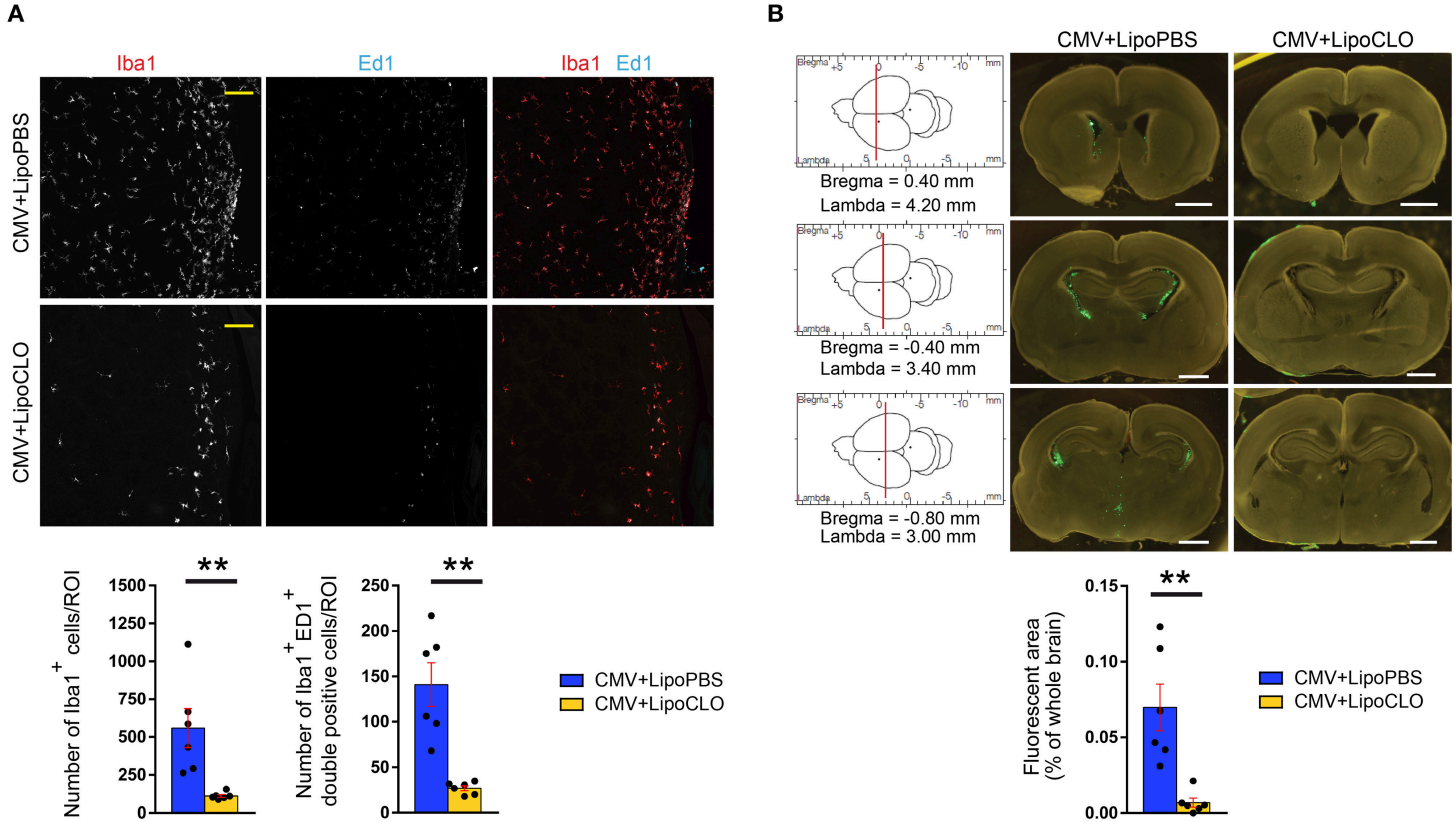
Clodronate liposomes deplete microglia and reduce CMV spreading in the developing brain. Recombinant rat CMV allowing expression of GFP (green) in the infected cells was injected icv in rat embryos at E15 together with either clodronate liposomes (LipoCLO) to deplete microglia, or PBS liposomes (LipoPBS) as control. **(A)** LipoCLO decreased the number of Iba1^+^ microglia (red) and of Iba1^+^, Ed1^+^ (cyan) phagocytically active microglia. Three to four adjacent coronal brain sections were analyzed by confocal microscopy throughout the entire z-dimension (*n* = 6 brains in each condition). ROI: region of interest (775 × 775 μm^2^). Bar: 100 μm. Mann-Whitney test, two-tailed, with Bonferroni correction. **p < 0.005. **(B)** LipoCLO reduced rat CMV infection of the brain. Brains were analyzed at P1 using fluorescent binocular microscopy. Three sections were selected according to their coordinates in the rostrocaudal axis (left). CMV infection decreased dramatically after treatment with LipoCLO (*n* = 6 brains in each condition), as quantified by measuring the proportion of fluorescent (GFP) areas. Bar: 1 mm. Mann-Whitney test, two-tailed. **p < 0.01.

In a first series of experiments, microglia were acutely depleted with clodronate liposomes co-injected icv together with rat CMV at E15. Immunohistochemistry experiments confirmed that clodronate liposomes triggered a significant decrease in the total number of Iba1^+^ microglial cells in the dorsolateral part of the striatal wall of the lateral ventricles taken as the region of interest (ROI) at P1 (112.3 cells/ROI ± 9.37), as compared to the condition where PBS liposomes were used (558.5 cells/ROI ± 128.3; *p* = 0.0022; Figure 3A; Supplementary Table 2). A significant decrease in the absolute number of phagocytically active, Iba1^+^ Ed1(CD68)^+^ microglia was also oberved (clodronate liposomes: 27.17 cells/ROI ± 2.80; PBS liposomes: 141 cells/ROI ± 23.82) (*p* = 0.0022). This was associated with a dramatic reduction of CMV spreading, since GFP^+^ infected cells were barely visible within the brains of treated embryos. The reduction in CMV infection was confirmed by quantifying the percentage of fluorescent areas in coronal brain sections taken from treated and untreated pups at P1 (*p* = 0.0022; Figure 3B; Supplementary Table 3).

Microglia depletion and reduction of brain CMV infection were associated with a significant improvement of survival and neurodevelopmental outcomes. Postnatal mortality was reduced by 2.9 fold (*p* = 0.012) in clodronate-treated, CMV-infected pups, relative to untreated, infected pups (Figures 1A, 2; Supplementary Table 1). Indeed, 69.6% (16 out of 23 newborns) of clodronate-treated, CMV-infected newborns survived at P16, as compared to 12.1% (4 out of 33) of untreated, CMV-infected newborns (*p* < 10^−4^, Chi2 test). A similar significant increase in body weight gain was observed in the clodronate-treated pups (*p* < 10^−4^; Figure 1B; Supplementary Table 1).

CMV-infected pups treated with clodronate liposomes *in utero* also performed significantly better at the righting and the cliff aversion reflexes when compared to infected pups, which had received control (PBS) liposomes *in utero* (Figures 1C,D, 2; Supplementary Figure 2; Supplementary Table 1). With clodronate the odds in favor of success to righting and to cliff aversion tests were at 20.1:1 and 18.4:1, respectively (*p* < 10^−4^ for both). Hindlimb paralysis also improved significantly (*p* < 10^−4^) with clodronate liposomes (17% of rat pups) as compared with control liposomes (88%) (Figures 1E, 2; Supplementary Table 1). There was a dramatic, 120.4-fold decrease in the risk to hindlimb paralysis in clodronate-treated CMV-infected rats (*p* < 10^−4^). Epileptic seizures also occurred less frequently in the first three weeks of life upon treatment with clodronate liposomes (8.7% of rats experiencing at least one seizure), as compared to PBS liposomes (24.2% of epileptic rats) but this difference did not reach statistical significance (*p* = 0.14) (Figures 1F, 2; Supplementary Table 1). However, when the risk of seizures was considered, it decreased significantly by 8.1-fold in clodronate-treated, CMV-infected rats when compared to their PBS-liposomes counterparts (*p* < 10^−4^).

Hence a single injection of clodronate liposomes at the time of CMV icv infection not only led to depletion of microglia and to a dramatic reduction of active CMV infection in the rat developing brain, but the treatment also led to a stunning improvement of survival, body weight gain, sensorimotor development and epileptic seizures in early postnatal life.

### Chronic administration of doxycycline to pregnant mothers improves microglia phenotype

Tetracyclines can efficiently modify microglia status in the brains of rat pups after chronic maternal administration during pregnancy (Cunningham et al., 2013). Tetracyclines impact on microglia phenotype independently of their canonical antibacterial action (Tikka et al., 2001). In order to confirm the possible role of microglia in the emergence of phenotypes associated with brain CMV infection, doxycycline, a second-generation, lipophilic tetracycline that crosses blood-brain and placental barriers, was administered *per os* to pregnant dams from E0 to birth. The effects of doxycycline treatment on microglia phenotype was tested by immunohistochemistry in the dorsolateral part of the striatal wall of the lateral ventricles, a region where active CMV infection was frequently detected (Cloarec et al., 2016), and by multicolor flow cytometry analysis of the whole brain.

**Figure 4:**
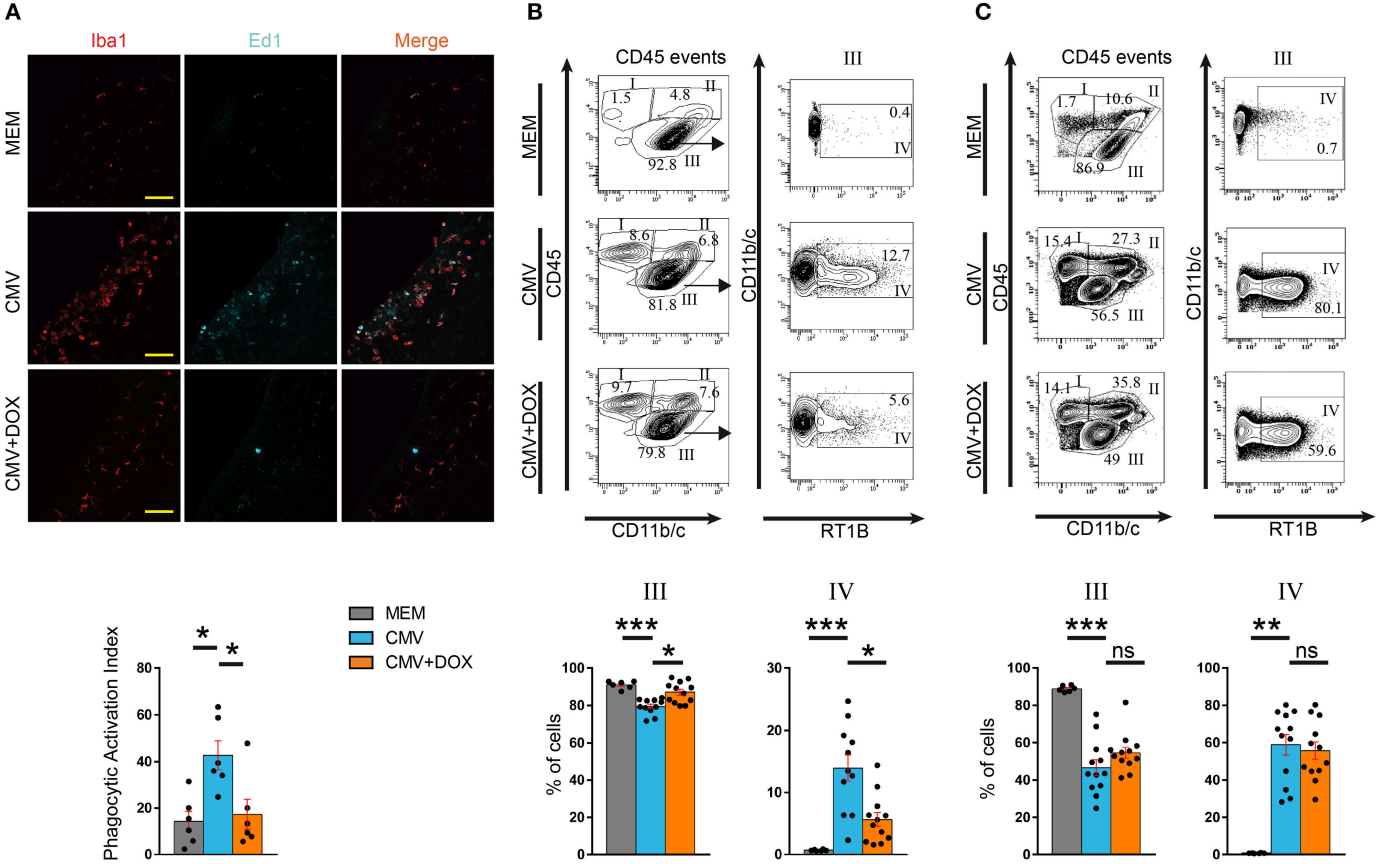
CMV infection of the embryonic rat brain leads to altered microglia phenotype, which is rescued by maternal feeding with doxycycline throughout pregnancy. **(A)** Microglia status in the lateral ventricles was assessed on coronal sections observed by confocal microscopy at P1 by quantifying microglial cells (red, Iba1^+^) and phagocytically active microglial cells (Iba1^+^, red; Cd68/Ed1^+^, cyan). Those values were then used to calculate the phagocytic activation index (PAI) defined as the ratio of the number of Iba1^+^, Ed1^+^ activated cells to the total number of Iba1^+^ cells. PAI increased in the CMV vs. the MEM group. Doxycycline (CMV+DOX) significantly counteracted PAI increase (*n* = 6 brains in each condition). ROI: region of interest (387 × 200 μm^2^). Bar: 50 μm. Kruskall-Wallis test followed by Dunn's *post-hoc* test. *p < 0.05. **(B,C)** Flow cytometry analysis of leukocytes collected at P1 **(B)** and P7 **(C)**. Total leukocytes (CD45 events) were gated for CD45 and Cd11b/c, thus defining fractions I (lymphocytic cells), II (myelo-monocytic cells) and III (resident microglial cells) (see also Supplementary Figure 3). Fraction III corresponding to CD45^low/int^, CD11b/c^+^ microglial cells was further characterized for RT1B expression to identify reactive microglia (RT1B^+^; fraction IV). Representative flow cytometry plots and the corresponding quantifications are shown for each group. **(B)** At P1, the increased proportion of reactive microglia triggered by CMV infection was significantly counteracted by doxycycline (*n* = 6 MEM; *n* = 11 CMV; *n* = 12 CMV+DOX). **(C)** At P7, i.e., 7 days after discontinuation of doxycycline, the decreased proportion of total microglial cells (fraction III) and, within that fraction, the increased proportion of reactive microglia (fraction IV) triggered by CMV infection, were not counteracted by doxycycline treatment given *in utero* (*n* = 6 MEM; *n* = 12 CMV; *n* = 12 CMV+DOX). Kruskall Wallis test followed by Dunn's post test. ***p < 0.001; **p < 0.01; *p < 0.05; ns, not significant.

In line with previously reported experiments (Cloarec et al., 2016), rat CMV infection at E15 led to a significant increase in the proportion of phagocytically active, Iba1^+^ Ed1(CD68)^+^ microglia/macrophages cells at P1. Indeed, the phagocytic activation index (PAI), defined as the ratio of Iba1^+^ Ed1^+^ cells to the total number of Iba1^+^ cells, was significantly increased (42.74% ± 6.16) as compared with control (MEM-injected) rats (PAI = 14.28% ± 4.29) (p = 0.0174), and was significantly improved by doxycycline (17.32% ± 6.44; *p* = 0.0321) (Figure 4A; Supplementary Table 2). Consistent data were obtained when whole brains were analyzed at P1 by flow cytometry (Figure 4B; Supplementary Table 4). As previously reported (Cloarec et al., 2016), a significant increase in the proportion of major histocompatibility (MHC) class II-positive microglia (fraction IV: CD45^low/int^, CD11b/c^+^, RT1B^+^) was detected in CMV-infected brains (13.98% ± 2.12) as compared with controls (0.73% ± 0.05; *p* < 0.0001). This increase in activated microglia was significantly counteracted in doxycycline-treated, CMV-infected pups (5.66% ± 1.13) (*p* = 0.0483). The proportions of other types of CD45^+^ hematopoietic cells were not changed by doxycycline (Figure 4B; Supplementary Figure 3; Supplementary Table 4). Importantly, the favorable impact of the doxycycline *in utero* treatment on reactive microglia seen at P1 did not last after treatment discontinuation. The proportion of reactive, CD45^low/int^, CD11b/c^+^, RT1B^+^ microglia (fraction IV) at P7 were similar in treated (55.76% ± 4.60) and in untreated (58.93% ± 5.54) CMV-infected rats (Figure 4C; Supplementary Figure 3; Supplementary Table 4).

**Figure 5:**
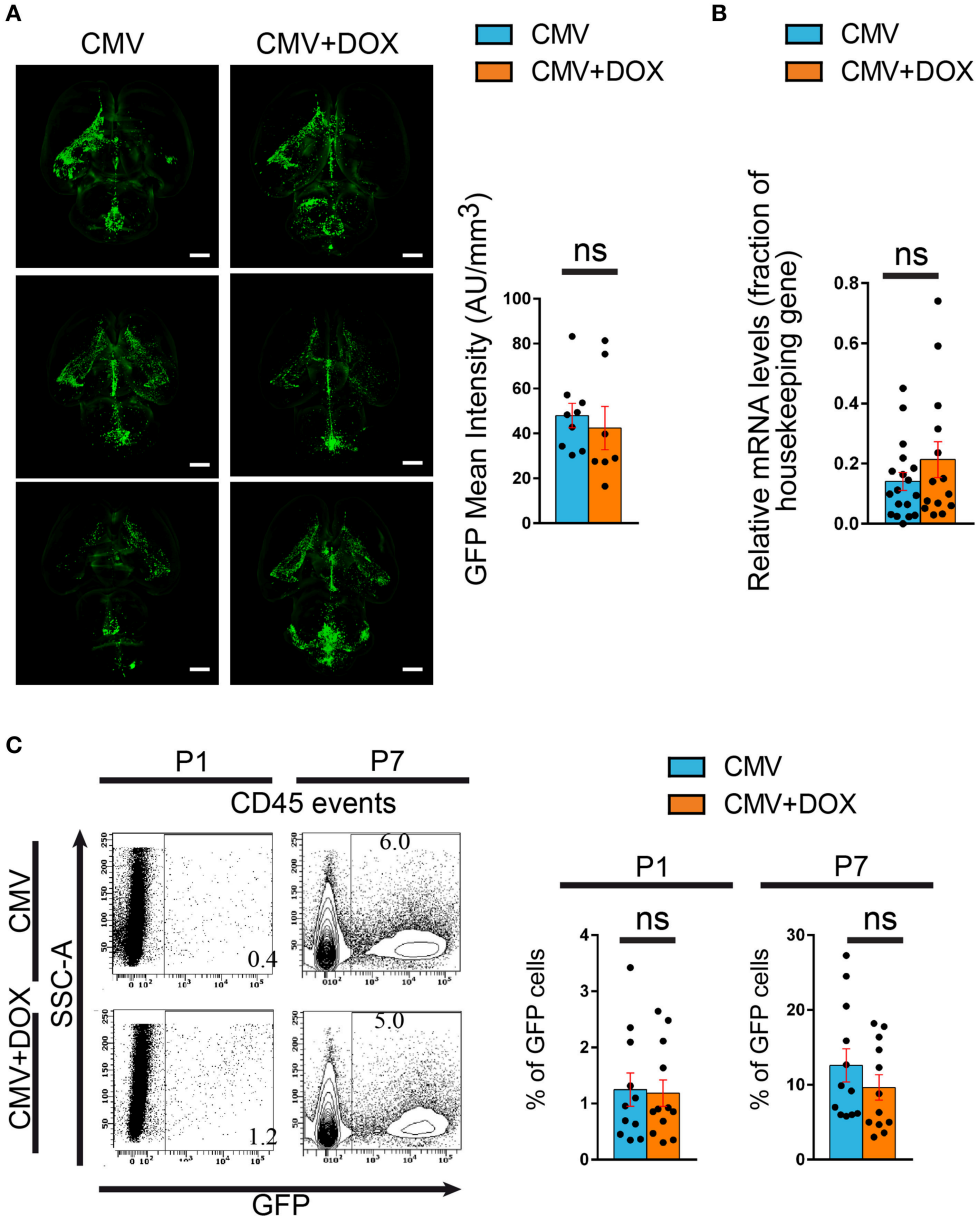
Maternal feeding with doxycycline throughout pregnancy does not impact rat CMV infection of the brain. Green fluorescent protein (GFP) expression was used to monitor infection of the pups' brains by rat CMV with three independent methods. **(A)** Tissue clearing. Whole brains from P1 rat pups were submitted to tissue clearing and GFP expression was quantified by measuring mean fluoresence intensity normalized to each corresponding total brain volume. Representative examples of clarified brains are shown (light sheet microscopy) (see also Supplementary Videos 1, 2). No significant difference was found between the CMV+DOX group (*n* = 7) and its untreated counterpart (CMV; *n* = 9). Bar: 2 mm. Mann Whitney test, two-tailed. ns, not significant. **(B)** qRT-PCR. Relative mRNA expression of GFP was assessed by quantitative RT-PCR in CMV-infected brains at P1, using *RPL-19* as reference gene. No difference was found between the CMV+DOX group (*n* = 14) and its untreated counterpart (CMV; *n* = 18). Values of fold change represent averages from triplicate measurements for each sample. Mann Whitney test, two-tailed. ns: not significant. **(C)** Flow cytometry. Relative proportion of GFP^+^, CMV-infected cells was estimated by flow cytometry analysis of CD45^+^ cells isolated from CMV-infected brains at P1 and at P7. No difference was found between the CMV+DOX group (*n* = 12) and its untreated counterpart (CMV; *n* = 11). SSC, side-scattered light. Mann Whitney test, two-tailed. Ns, not significant.

The consequences of *in utero* treatment with doxycycline on brain CMV infection *per se* were also evaluated. Independent series of brains were submitted to tissue clearing using the iDisco method (Renier et al., 2014). Fluorescence-based 3D quantification of the infected areas of whole brains showed no difference between treated and untreated rats at P1 (Figure 5A; Supplementary Table 3; Supplementary Videos 1, 2). Consistently, no significant difference in GFP gene expression was found by qRT-PCR between treated and untreated CMV-infected brains at P1 (Figure 5B; Supplementary Table 5). Moreover, the proportions of CMV-infected, GFP^+^ cells as detected by flow cytometry analysis performed on CD45^+^ hematopoietic cells isolated from CMV-infected brains at P1, were not significantly different between doxycycline-treated and untreated pups (Figure 5C; Supplementary Table 4). Also, no significant difference in GFP^+^ infected cells was found by flow cytometry at P7 between doxycycline-treated and untreated pups. Hence the early and transient impact of doxycycline on microglia phenotype was not accompanied by a parallel decrease in CMV infection of the brain.

### Chronic doxycycline administration to the mother throughout pregnancy improves the postnatal outcome

Owing to the transient improvement of microglia status upon maternal administration of doxycycline during pregnancy, we then tested whether this would in turn impact the postnatal outcomes. In doxycycline-treated, CMV-infected pups, survival rate improved significantly as compared to infected pups from untreated dams (Figure 1A, 2; Supplementary Table 1). CMV-infected pups treated with doxycycline *in utero* had a risk 5.1 lower to postnatal death in the first three postnatal weeks, than their untreated, CMV-infected counterparts (*p* = 0.0006). At P20, 86.1% (*n* = 31 out of 36 newborns) of infected pups from doxycycline-treated dams had survived as compared to 29.6% (*n* = 16 out of 54 newborns) of infected pups from untreated dams (*p* < 0.001, Chi2 test). Significant improvement in body weight gain was also observed in CMV-infected pups after doxycycline treatment *in utero* (*p* < 10^−4^; Figure 1B; Supplementary Table 1).

Whereas no rescue could be obtained at P40 on hearing threshold after doxycycline treatment (Supplementary Figure 1; Supplementary Table 1), responses to sensorimotor tests (righting and cliff aversion reflexes) improved significantly (*p* = 0.025 and *p* < 10^−4^, respectively) in infected pups treated with doxycycline *in utero* (success to righting: odds ratio 2.6:1; success to cliff aversion: odds ratio 9.9:1) (Figures 1C,D, 2; Supplementary Figure 2; Supplementary Table 1).

Hindlimb paralysis was also observed less frequently after doxycycline administration (Figures 1E, 2; Supplementary Table 1). Hence, 50% (*n* = 18 out of 36) of doxycycline-treated, CMV-infected pups displayed paralysis during the first three postnatal weeks, as compared to 83.3% (*n* = 45 out of 54) in the untreated counterparts (*p* = 0.001). Consistently, the risk of hindlimb paralysis reduced by 6.2 fold (*p* = 0.03). Whereas the proportion of epileptic, CMV-infected rats decreased, albeit not significantly (*p* = 0.12), upon treatment with doxycycline (11%) as compared with untreated, infected rats (24%), the risk of epileptic seizures during the period of evaluation decreased significantly by 5.9-fold in CMV-infected rats receiving doxycycline *in utero* (*p* < 10^−4^; Figures 1F, 2; Supplementary Table 1).

Hence, while doxycycline administration during pregnancy did not significantly modify the amount of active CMV infection in the developing brain after birth, it led to a transient modification of microglia phenotype that was associated with long-term favorable effects on survival, body weight gain and neurodevelopmental outcomes.

## Discussion

### Phenotypes in the rat and in humans

Studies on different rodent models that showed similarities with the human disease at the neuroanatomical, cellular and molecular levels, have suggested that neuroimmune alterations might play an important pathophysiological role. In a rat model of CMV infection of the developing brain *in utero*, we reported recently the detection of early neuroimmune anomalies including microglia alteration (Cloarec et al., 2016). Utilizing this model, we herein demonstrate altered postnatal oucomes in CMV infected neonates including increased postnatal lethality, decreased body weight gain, early-onset epileptic seizures, sensorimotor impairment reminiscent of cerebral palsy, and hearing defects. Despite the fact that maternal, placental and peripheral embryonic events were purposedly bypassed by directly infecting the embryonic brain *in utero*, this model recapitulated several fundamental phenotypic features of the human pathology following CMV infection. Moreover, the postnatal outcomes varied dramatically among the rat pups, as in the human disease. This indicates that in addition to the aforementioned upstream factors, the somehow unpredictable and highly diverse outcome of congenital CMV infection might rely, at least partly, on fetal brain-related events. The consequences of CMV infections of the brain on the future neurological phenotypes had been hardly addressed in rodent models until recently. Auditory features associated with inflammation of the inner ear recalling the hearing losses seen in human congenital infections were recently reported in a neonatal mouse model (Bradford et al., 2015). Also, early and long-term neurological dysfunctions including acquisition of the righting and cliff aversion reflexes as well as motor performances and social behavior have been reported very recently in a murine model of neonatal infection (Ornaghi et al., 2017). Interestingly, in the present rat model of rat CMV infection *in utero*, more dramatic neurological consequences were observed, probably because CMV was inoculated directly into the ventricles and at an earlier developmental stage.

### Targeting microglia with doxycycline and clodronate

Generally, microglia-targeted rescue strategies had already been successfully used in a range of models for various postnatal neurological insults and disorders, such as neonatal excitotoxic brain damage (Dommergues et al., 2003), experimental autoimmune encephalitis (Ponomarev et al., 2011), cerebral palsy (Kannan et al., 2012), or Alzheimer disease (Hong et al., 2016). In the present model of prenatal cerebral infection, two previously reported microglia-targeted drug-based strategies both led to the successful rescue of the neurological phenotypes. While the respective modes of action of clodronate liposomes on the one hand, and doxycycline on the other hand, are unrelated at the molecular and subcellular levels, we cannot firmly exclude the possibility that they would both have influenced the phenotypes by microglia-independent, off-target mechanisms. Doxycycline might have other biological consequences (Yrjanheikki et al., 1999) such as a broader anti-inflammatory action on other immune and non-immune cells (Yrjanheikki et al., 1998). Hence, it was shown in a murine model of multiple sclerosis that minocycline, another second-generation tetracycline, can decrease leukocytes infiltration into the spinal cord (Brundula et al., 2002). However, in our model doxycycline did not modify the different fractions corresponding to the non-microglial immune cells detected in the CMV-infected brains (e.g., monocytes/macrophages, dendritic cells, T cells and B cells), as analyzed by flow cytometry (see Supplementary Figure 3). Moreover tetracyclines have already been used to decrease microglia activation in NMDA-induced excitotoxicity (Tikka et al., 2001), experimental autoimmune encephalitis (Popovic et al., 2002), or epileptogenesis (Abraham et al., 2012).

### Influence of early microglia alteration on the postnatal phenotypes

Whereas clodronate liposomes led to microglia depletion associated with a dramatic decrease in brain CMV infection, doxycycline promoted microglia modification while leaving CMV infection of the brain unchanged. Moreover, the early improvement of microglia was associated with long-term beneficial effects. Indeed, the effect of doxycycline on microglia was transient and was no longer detected at P7. Despite this observation, the favorable impact on the clinical phenotypes lasted long after treatment was stopped. This suggests that the early alteration of microglia following CMV infection had long-term detrimental effects on the neurological and other outcome. Although we cannot exclude the existence of more subtle changes such as a modification in the distribution of CMV in different brain areas, our data indicate that early microglia alteration, rather than CMV infection and viral cytopathic effects *per se*, promoted the emergence of most neurological and other postnatal manifestations. The relationship between CMV load and the occurrence and severity of CMV-related phenotypes has indeed been challenged in a murine model of neonatal CMV infection (Kosmac et al., 2013; Seleme et al., 2017) and in human congenital CMV (Revello et al., 1999; Boppana et al., 2013). Our data are congruent with recent reports on inflammatory processes in CMV infection of the murine neonatal brain (Kosmac et al., 2013; Bradford et al., 2015; Kawasaki et al., 2015; Slavuljica et al., 2015; Seleme et al., 2017).

The microglia-targeted strategies used in the present study led to significant improvements of sensorimotor impairments and of epilepsy. The alteration and possible role of microglia in epileptic disorders and in cerebral palsy have already been discussed (Fleiss and Gressens, 2012; Vezzani et al., 2012; Devinsky et al., 2013). The presence of hearing impairment in the present model might be difficult to interpret. First, a progressive deterioration of auditory performances was seen in the control rats; this is consistent with previous reports on the existence of age-related hearing loss in control Wistar rats (Alvarado et al., 2014), which was likely related with the lack of strial melanin in albino rodents (Ohlemiller, 2009). Second, a detrimental effect of doxycycline on the development of the auditory system cannot be excluded, notably given its known potential auditory toxicity. Third, whereas hearing loss caused by murine neonatal CMV infection was recently associated with persistent inflammation of the inner ear (Bradford et al., 2015), the lack of significant improvement after doxycycline treatment *in utero* might indicate that microglia does not play an important role in hearing impairment in our model.

### Microglia in brain development and in neurodevelopmental disorders

A crucial pathogenic impact of early microglia alteration in the context of congenital CMV is consistent with the role of microglia in the development, the functioning and the pathology of the central nervous system (Prinz and Priller, 2014). Microglia undergo several programmed changes during brain development in rodents (Matcovitch-Natan et al., 2016), impact synaptic transmission, as well as synapse formation and elimination. Microglia also shape embryonic and postnatal brain circuits (Paolicelli et al., 2011; Schafer et al., 2012). The alteration of microglia by CMV infection early during brain development might well have disrupted the timing of such developmental programs, leading to altered development of neuronal networks and the subsequent detrimental neurological outcome. As an example, susceptibility to epileptic seizures could be caused by several microglia-dependent processes, such as the alteration of migration and neocortical laminar distribution of interneurons, the altered control of synaptic development and regulation, or an increased glutamate release (Paolicelli and Ferretti, 2017). As a matter of fact, the transient improvement of microglia status as obtained here with doxycycline was sufficient to obtain dramatic improvements of the neurological phenotypes. Prenatal alteration of microglia and the accompanying microglia priming can have long-term consequences on synaptic functioning (Roumier et al., 2008) and could participate in the susceptibility to neurodevelopmental disorders occurring as consequences of viral infections of the developing brain.

### Besides microglia

Whereas early microglia improvement was sufficient to impact significantly the prenatal phenotypes, this of course does not exclude the important roles likely played in parallel by other components of the neuroimmune system, either at the molecular level such as cytokines and chemokines, or at the cellular level such as infiltrating monocytes or lymphocytes. Hence, the role of T lymphocytes infiltration in CMV infection has been proposed (Bantug et al., 2008); T cells control viral spread within the brain and are pivotal in resolving acute CMV infection of the neonatal murine brain (Slavuljica et al., 2015). However, the amount of T cells was not impacted by doxycycline in the present model, which is consistent with the lack of any significant change in CMV infection observed here after doxycycline treatment. Similarly, doxycycline did not modify the proportions of dendritic cells or monocytes within the infected brains. Other types of glial cells such as astrocytes are also likely to play a pathophysiological role, as reported in other models of CMV infection (Lokensgard et al., 2016; Ornaghi et al., 2017), and notably given the known reciprocal interactions between microglia and astrocytes.

### Besides CMV

Apart from congenital CMV, microglia infection and alteration can occur in a wide range of viral encephalitis. Microglia might participate in neuronal damage seen in encephalitis caused by Japanese encephalitis virus (Thongtan et al., 2012). Microglia status might inform on neuronal dysfunctioning following murine leukemia virus-induced encephalopathy (Xue et al., 2010). Microglia might also participate in HIV-associated cognitive and behavioral impairments (Hong and Banks, 2015). Il34^−/−^ mice with fewer microglia were protected from synaptic defects triggered by West Nile virus infection (Vasek et al., 2016). Interestingly also, acute administration of minocycline prevented against long-term behavioral outcome in a model of temporal lobe epilepsy caused by infection with Theiler's virus (Barker-Haliski et al., 2016). Whether microglia-targeted strategies could be successfully applied to these and other congenital infections of the brain remains to be demonstrated.

### From the rat model to the human disease

Although extrapolation to the corresponding human pathology should be approached with caution, it is tempting to speculate that microglia might also play an important role in human neurological disease following congenital CMV infection. As a matter of fact, microglial nodules and infected microglial cells were detected in human CMV-infected fetal brains (Teissier et al., 2014). While different innovative strategies to combat against CMV infection and its consequences have been proposed recently in complementary rodent models (see Ornaghi et al., 2017), microglia represent another promising target for various neuropathological conditions (Cartier et al., 2014) Strategies directed toward the immune system have already been proposed with the use of corticosteroids in a mouse model of neonatal CMV infection (Kosmac et al., 2013) and with the administration of immunoglobulins to prevent against human CMV congenital infection (Revello et al., 2014). Pharmacological interventions *in utero* have already been successful in rodent models of motor impairment (Yamada et al., 2009) and of epilepsy (Salmi et al., 2013). The use of doxycycline in pregnancy could also be revisited in line with its potential benefits (Cross et al., 2016). Generally risk reduction of fetal infection and subsequent disease still remains a crucial issue (Hamilton et al., 2014). Together with progress in fetal medicine (Bianchi, 2012) and in the identification of reliable biomarkers to predict severity (Desveaux et al., 2016), the present study, which now warrants confirmation and expansion using complementary rodent models of CMV infection and other microglia modifying tools, represents a first proof-of-principle for the future design of microglia-targeted strategies to prevent against the severe neurological outcome of congenital CMV infection of the brain.

## Concluding remarks

In conclusion, we have shown here that the neurological and other phenotypes caused by CMV infection of the rat brain *in utero*, are caused by the early activation of microglia, rather than by the virus load by itself, at least in this model - suggesting that microglia might also influence the corresponding human disease.

## Author contributions

RC and SB performed most experiments and data analyzes, with equal contribution. NT supervised, performed and validated auditory tests and participated in the overall strategy. FS performed most *in utero* injections. HL supervised and participated in flow cytometry. SC performed a subset of immunohistochemistry, confocal microscopy and image analyzes. MS participated in immunohistochemistry and pilot rescue experiments at the initial stages of the project. VP designed and performed most statistical analyzes. EBo performed and analyzed auditory tests. EP-P performed and analyzed qRT-PCR. EBu supervised *in utero* injections and performed a subset of them. FM headed the InMAGIC imaging platform and participated in images acquisition and analyzes. PG participated in the follow-up of the project and in the overall strategy. MM provided strong scientific support on flow cytometry analyzes. TS and DS provided and purified the RCMV strain and provided strong expertise in virology. NB provided scientific support and advices all along the duration of the project and participated in the overall strategy. PS was the project leader. He decided on the overall strategy, directed the follow-up of experiments, supervised data analysis, and wrote the manuscript with help of RC and SB. All authors contributed the final version of the manuscript.

### Conflict of interest statement

RC has been a recipient of an INSERM/PACA Ph.D. fellowship and a recipient of a FRM Ph.D. fellowship (FDT20140930813), during which he performed all his experiments and data analyzes except the iDisco experiments, which he performed and analyzed while being a Neurochlore employee. A patent application has been filed and submitted to the European Patent Office via the Tech Transfer office at INSERM. The other authors declare that the research was conducted in the absence of any commercial or financial relationships that could be construed as a potential conflict of interest.
