# Supplementary material for: In Utero Administration of Drugs Targeting Microglia Improves the Neurodevelopmental Outcome Following Cytomegalovirus Infection of the Rat Fetal Brain
Source: Front Cell Neurosci. 2018 Mar 6;12:55. doi: 10.3389/fncel.2018.00055 (PMC5845535; doi:10.3389/fncel.2018.00055)
Supplement: Supplementary file 6 [file Image1.pdf]

Supplementary Figures

In utero administration of drugs targeting microglia improves the neurodevelopmental outcome following cytomegalovirus infection of the rat fetal brain

Robin Cloarec[1,2]*, Sylvian Bauer[1]*, Natacha Teissier[3,4], Fabienne Schaller[1,5], Hervé Luche[6], Sandra Courtens[1], Manal Salmi[1], Vanessa Pauly[7], Emilie Bois[3,4], Emilie Pallesi-Pocachard[1,8], Emmanuelle Buhler[1,5], François J. Michel[1,9], Pierre Gressens[3,4], Marie Malissen[6], Thomas Stamminger[10], Daniel N. Streblow[11], Nadine Bruneau[1], Pierre Szepetowski[1]¶

*both authors contributed equally to this work

[1]INMED, Aix-Marseille University, INSERM U1249, Marseille, France. [2]Neurochlore, Marseille, France. [3]INSERM U1141, Paris Diderot University, Sorbonne Paris Cité, Paris, France. [4]PremUP, Paris, France. [5]PPGI platform, INMED, Marseille, France. [6]CIPHE (Centre d'Immunophénomique), PHENOMIN, Aix-Marseille University, INSERM US012, CNRS UMS3367, Marseille, France. [7]Laboratoire de Santé Publique EA 3279, Faculté de Médecine Centre d'Evaluation de la Pharmacodépendance-Addictovigilance (CEIP-A) de Marseille (PACA-Corse) Associé, Aix-Marseille University, Marseille, France. [8]PBMC platform, INMED, Marseille, France. [9]InMAGIC platform, INMED, Marseille, France. [10]Institute for Clinical and Molecular Virology, University of Erlangen-Nuremberg, Erlangen, Germany. [11]Vaccine & Gene Therapy Institute, Oregon Health and Science University, Portland, OR, USA.

¶Correspondence to: Dr Szepetowski, Institut de Neurobiologie de la Méditerranée (INMED), Inserm U1249, Parc Scientifique de Luminy, BP13, 13273 Marseille Cedex 09, France. Phone: +33 (0)4 9182 8111; Fax : +33 (0)4 9182 8101; e-mail: pierre.szepetowski@inserm.fr

Supplementary Figure 1. Hearing threshold evaluation.

For auditory experiments, evoked potentials were performed at P40 in three groups of rats: previously subjected to either of the following procedures during pregnancy: injection of minimal essential medium (MEM) intraventricularly (icv) at embryonic day 15 (E15) (control group, MEM; n = 12 from two litters); injection of rat CMV icv in pregnant rat fed with doxycycline (DOX) *per os* all over pregnancy (DOX-treated infected pups, CMV+DOX; n = 25 from five litters); injection of rat CMV icv (untreated - no DOX - infected pups, CMV; n = 11 from four litters). Four needle electrodes were placed subcutaneously under 1.5 % isoflurane anesthesia. For each rat, the reference electrode was inserted beneath the pinna of the assessed ear, the positive electrode beneath the skin on the vertex of the head, the ground electrode on the animal's back. Evoked potentials were performed using the Echodia® (Saint-Beauzire, France) apparatus and the RTlab software. Headphones with appropriate earplugs were used as acoustic transducers. Clicks, 12 kHz and 24 kHz tone bursts were delivered at a frequency of 17 Hz. Filters were set at 150-1500 Hz. Rejection threshold was defined at 20 µV. Impedances were monitored to be below 2000 Ω. Responses for 250 sweeps were averaged at each intensity level. The stimulus intensity was decreased by 10 dB steps sound pressure level (SPL) alternating right and left ears. A contralateral auditory masking was used for high intensity stimulations (> 45 dB). Thresholds were defined as the lowest level at which a reproducible wave IV response could be obtained; the curves were analyzed by two different researchers, of whom one was blinded to the study. Profound hearing loss and cophosis were defined by the absence of reproducible wave IV at 90dB (at 80dB for the 12 kHz tone bursts); if no threshold was identified at 90 dB, the recording was repeated on the following day to exclude any technical problem. For statistics' sake, these thresholds were set at 100 dB. Of note, no significant difference in sex ratio was observed between the three groups (p = 0.37, Chi-square test). Clicks (A), 12 kHz (B) and 24 kHz (C) tone bursts were delivered and hearing thresholds (decibels, dB) were determined. The CMV group showed significantly higher thresholds for clicks (C) and at 24 kHz (E) as compared with the MEM group. Doxycycline did not rescue hearing impairment. Kruskall Wallis test followed by Dunn's post test: ***: p < 0.001; **: p < 0.01; ns: not significant.

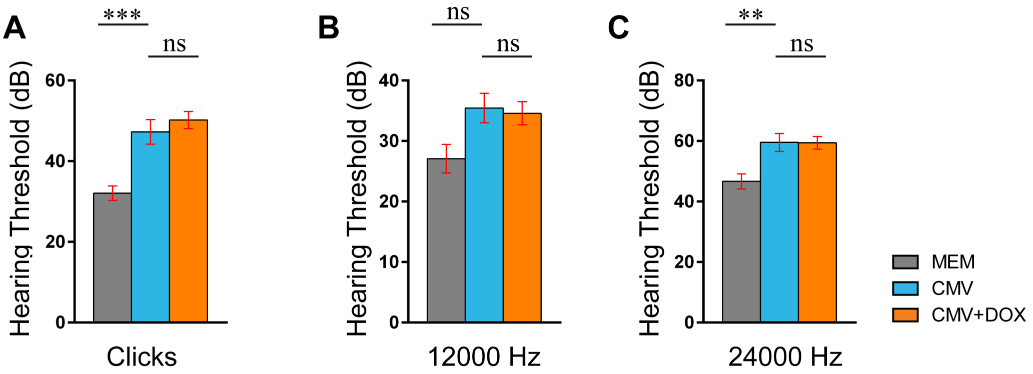

Supplementary Figure 2. Daily analyses of times to succeed to the righting reflex and to the cliff aversion tests.

Times to perform the righting reflex (A) and the cliff aversion reflex (B) sensorimotor developmental tests were determined in the five cohorts of the subset of rat pups who succeeded (*i.e*. times < 30 sec., see Methods: for both tests, a maximum observation time of 30 sec. was used).

(A) When only the pups who succeeded to the test were compared, time required to right successfully did not differ significantly between the CMV and the MEM groups. Time required to succeed the righting reflex test decreased significantly in clodronate liposomes-treated (LipoCLO) pups vs PBS liposomes-treated pups (LipoPBS). No significant difference in the time to succeed was observed after doxycycline treatment (CMV+DOX) *vs* untreated condition (CMV).

(B) When successful, CMV-infected pups needed more time to turn away from the edge than control (MEM) pups. Time required to succeed the cliff aversion reflex test decreased significantly in clodronate liposomes-treated (LipoCLO) pups vs PBS liposomes-treated pups (LipoPBS). Time to perform successfully the cliff aversion test was significantly decreased after doxycycline treatment (CMV+DOX) as compared with the untreated condition (CMV).

Mixed model for repeated data was used for statistical analysis: ***: p < 0.001; **: p < 0.01; *: p < 0.05; ns: not significant.

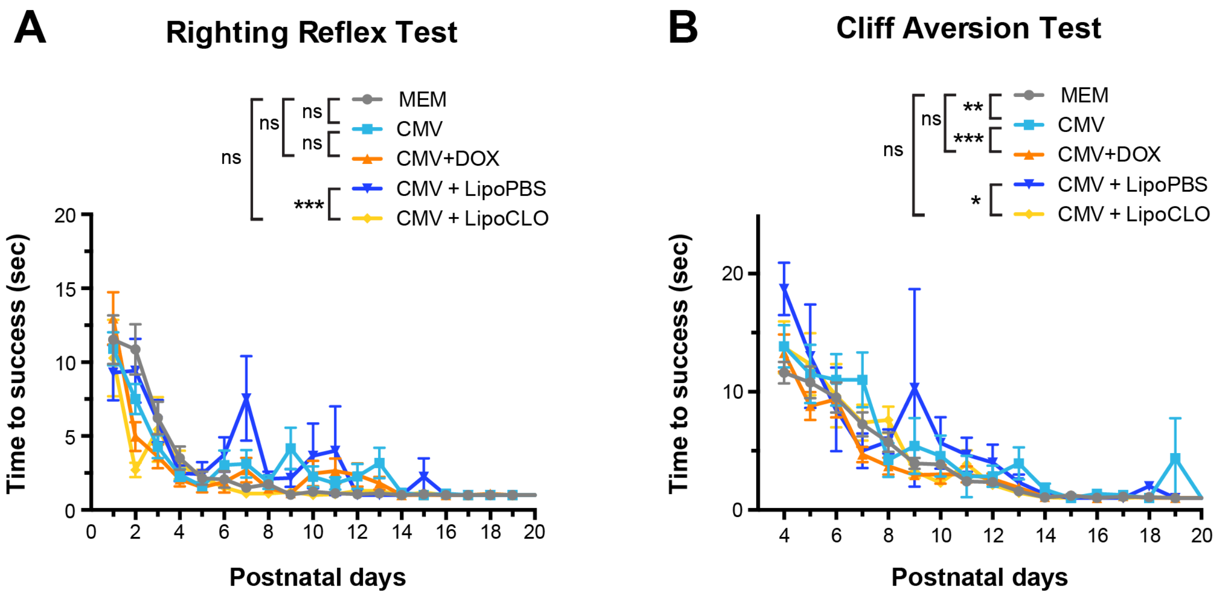

Supplementary Figure 3. Complementary data of flow cytometry analyses of leukocytes subpopulations at P1 after doxycycline treatment.

Total leukocytes (CD45 events) were gated for CD45 and either Cd11b/c or CD11b expressions and further characterized for CD3 and RT1B or for CCR2 expressions, respectively. Apart from microglia (see Fig.4B), the proportions of fraction I cells ($CD45^{high}$ $CD11b/c^{-}$) corresponding to the lymphoid lineage (CMV+DOX: 5.70% ± 0.99; CMV: 9.18% ± 1.12) and, within this fraction, of T cells (CMV+DOX: 0.45% ± 0.06; CMV: 0.69% ± 0.10), B cells (CMV+DOX: 0.67% ± 0.14; CMV: 1.31% ± 0.26) and $RT1B^{-}$ $CD3^{-}$ cells (CMV+DOX: 4.57% ± 0.82; CMV: 7.19% ± 0.81), did not change significantly upon doxycycline treatment. Similarly, the proportion of fraction II cells ($CD45^{high}$ $CD11b/c^{+}$) corresponding to the myeloid lineage (CMV+DOX: 5.15 ± 0.59; CMV: 4.98% ± 0.49) and, within this fraction, of dendritic cells (CMV+DOX: 1.76% ± 0.25; CMV: 2.34% ± 0.30) and of monocytes (CMV+DOX: 1.34% ± 0.20; CMV: 2.47% ± 0.60), did not change significantly either after doxycycline treatment.

(top) Representative flow cytometry plots in control (MEM-injected), CMV-infected (CMV), and doxycycline-treated, CMV-infected (CMV+DOX) brains. Fraction I (CD45hi CD11b/c-) corresponds to lymphocytes/natural killers and non-B non T cells. Fraction II (CD45hi CD11b+) corresponds to monocytes/macrophages, monocytes-derived dendritic cells and granulocytes. Fraction IIb/c (CD45hi CD11b/c+) corresponds to monocytes/macrophages, myeloid dendritic cells and granulocytes.

(bottom) The distribution of fraction I, and within this fraction, of T-lymphocytes and of RT1B- CD3- cells, was significantly different in CMV-infected brains as compared with control (MEM) brains. The proportions of fraction I cells and, within this fraction, of T cells, B cells and $RT1B^{-}$ $CD3^{-}$ cells did not differ significantly between the CMV+DOX and the untreated CMV cohorts. The proportions of fraction II cells and, within this fraction, of dendritic cells and of monocytes did not differ significantly either between the CMV+DOX and the untreated CMV cohorts.

Analyzes were performed using brains from control pups (MEM; n = 6), untreated CMV-infected pups (CMV; n = 11), and doxycycline-treated CMV-infected pups (CMV+DOX; n = 12). Values are means ± SEM. The statistical significance of the observed variation in frequency in each cell population is indicated. Kruskall Wallis test followed by Dunn's post test. ***: $p < 0.001$; **: $p < 0.01$; *: $p < 0.05$; ns: not significant.

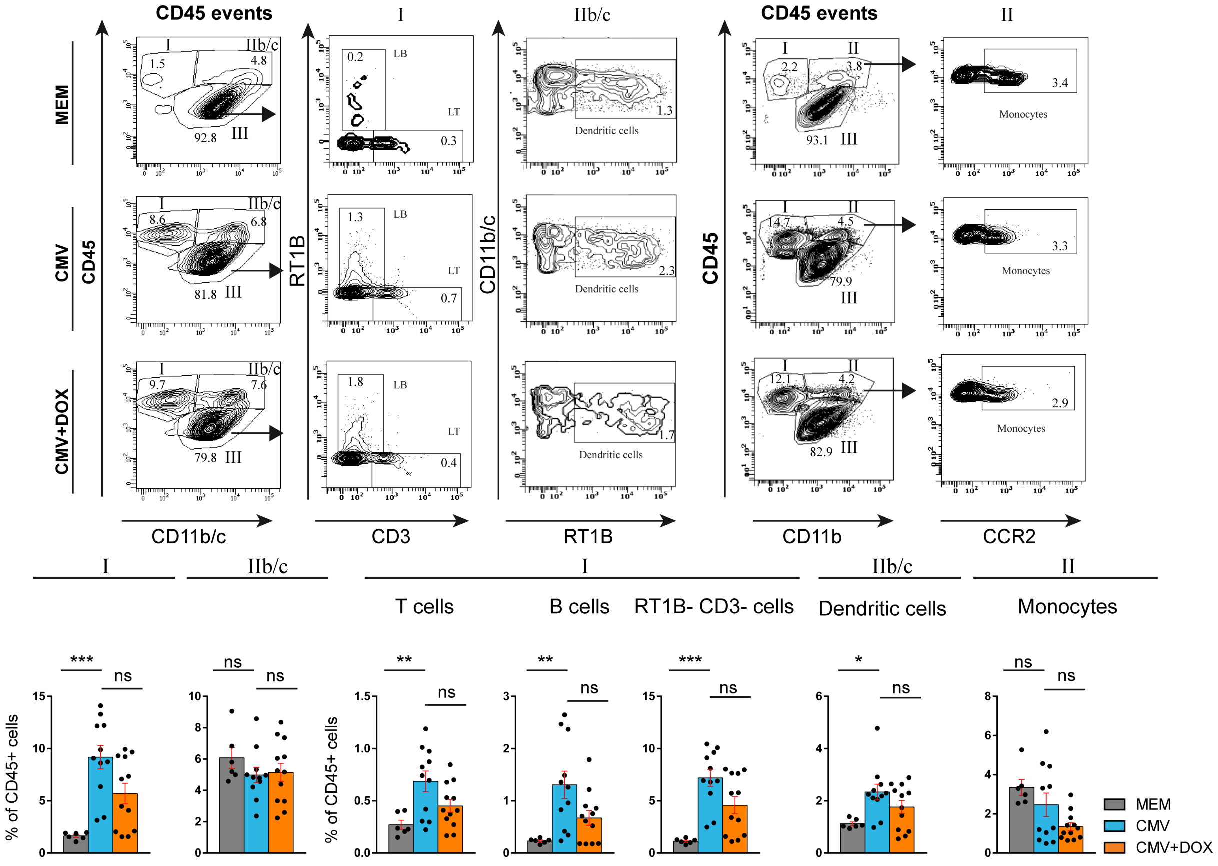

CD45 events
I
IIb/c
CD45 events
II
MEM
CMV
CMV+DOX
CD45
RT1B
CD11b/c
CD45
CD11b/c
CD3
RT1B
CD11b
CCR2
LB
LT
Dendritic cells
Monocytes
T cells
B cells
RT1B- CD3- cells
Dendritic cells
Monocytes
% of CD45+ cells
***
**
*
ns
MEM
CMV
CMV+DOX
